# Does Love Influence Athletic Performance? The Perspectives of Olympic Athletes

**DOI:** 10.5539/res.v8n2p1

**Published:** 2016-03-09

**Authors:** Kelly Campbell, Cheyenne Hosseini, Kelly Myers, Nina Calub

**Affiliations:** 1 Department of Psychology, College of Social and Behavioral Sciences, California State University, San Bernardino, USA

**Keywords:** athletic performance, couple relationship, cross-cultural conceptualization, love, Olympic athlete, passion

## Abstract

In this brief report, we provide an initial account of the association between love and athletic performance from the perspective of Olympic athletes. We posit that Romantic Passionate Love (RPL) and athletic performance may both involve the reward-motivation system of the brain. Based on this premise, we explored whether activation in one domain (love) might influence the other (sport). Our investigation was framed using Sternberg’s triangular theory of love. Twenty Olympic athletes representing different sports were interviewed at the Games. Most athletes (n = 15) reported that their performance was better while in love; however, qualitative responses suggested that the benefits were correlated with rather than resulting from RPL. Although the athletes were provided with a definition of RPL and affirmed that their relationship met the criteria, interview responses reflected companionate rather than passionate love, suggesting that RPL may be differentially conceptualized across cultures. The study provides preliminary data that may be used to inform and refine future work on this topic.

## 1. Introduction

Romantic-Passionate Love (RPL), or being “in love” is a euphoric state experienced in relationships cross-culturally. It is characterized by exhilaration, obsessive thoughts, and increased energy (Aron, Fisher, Mashek, Strong, Li, & Brown, 2005). The state of vitality that characterizes RPL may either detract from, or enhance an individual’s performance in various activities. For example, researchers who examined RPL and creativity found that creativity was enhanced during this relationship stage (Campbell & Kaufman, 2015). Because of the direct association between RPL and the reward-motivation system of the brain (Aron et al., 2005), RPL may influence behaviors associated with this brain region. In other words, the effect of RPL, whether positive or negative, may be pronounced for individuals who are already focused on tasks associated with the reward-motivation system. Our research extends the literature on the effects of RPL by examining its association with athletic performance. RPL and athletics are characterized by overlapping qualities such as strong emotion, focused attention, and high energy, which may either enhance or detract from a person’s experience in each domain. Given that prior work has not focused on this topic, our study is exploratory and will provide an account of the connection between love and athletic performance using self-report methods. We begin with a review of the literature on love, including what it means to be “in love”.

### 1.1 Triangular Theory of Love

Sternberg’ (1986; 1998) Triangular Theory explains love according to three components: Passion, intimacy, and commitment. *Passion* involves strong sexual attraction. It is the least controllable component and the most likely to decline over time. In the early phases of a relationship, passion reaches its peak due to the positive drive of arousal (Lemieux & Hale, 2002). With time, the negative drive associated with decreasing arousal develops, creating equilibrium and stability (Sternberg, 1998). *Intimacy* is characterized by feelings of closeness and connectedness. Partners with high intimacy tend to have a trusting, affectionate bond. *Commitment* refers to a conscious decision to love a partner and maintain a relationship. This component is characteristic of long-term partnerships, although in arranged marriages, it may be the first to develop.

Passion, intimacy, and commitment combine to create different love-types. Two types that form the basis of most long-term relationships in North America are RPL and companionate love. RPL refers to being “in love” and is characterized by high passion and intimacy. This love-type tends to exist during the first few years of a romantic relationship. Due to the passion component, RPL is vulnerable to decline and partners who do not develop the commitment component risk breakup once their passion fades. Companionate love is defined by high levels of intimacy and commitment. This love-type is more stable because it is based on shared interests, trust, and deliberate maintenance of the relationship. In this paper, we focus on RPL because of its demonstrated association with the reward-motivation system of the brain (Aron et al., 2005), which we believe may correspond with the domain of athletics.

### 1.2 Biological Correlates of Love

Consistent with Sternberg’s theory, Fisher (2006) described three brain systems associated with love: Sex drive, attraction (RPL), and attachment (companionate love). Given that this work is rooted in evolutionary psychology, Fisher also identified a function for each love component. Sex drive is defined by a yearning for sexual gratification and is strongly influenced by estrogens and androgens. Its purpose is to provide motivation for engaging in sexual intercourse in order to maximize reproductive fitness. Attraction refers to the euphoric state of RPL and includes increased energy, focused attention, and intrusive thinking about a specific partner. These characteristics are influenced by high levels of catecholamine, dopamine, norepinephrine, and low levels of the indoleamine, serotonin (Fisher, 2006; Fisher, Aron, & Brown, 2005). The purpose of RPL is to focus attention on a specific person in order to save time and energy when selecting amongst mating partners. Attachment or companionate love is categorized by a calm, secure, and emotional union between two people. This state is influenced by the neuropeptides of oxytocin and vasopressin. Attachment evolved to help individuals remain attached to their partners long enough to rear offspring (Fisher, 2006).

In a test of this theory, Aron et al. (2005) examined the brain regions associated with love and found that individuals in the RPL stage experience activation in the dopamine opulent, reward-motivation regions of the brain. Study participants who were presented with a picture of their beloved while in an MRI scanner exhibited oxygenated blood flow in the right ventral midbrain around the VTA, dorsal caudate body, and caudate tail, which was significantly more noticeable compared to the control groups (Aron et al., 2005). The researchers noted that the euphoric state associated with RPL motivates individuals to remain in this stage because of the aforementioned brain activity, which causes a craving for attention from and intrusive thinking about the RPL partner.

### 1.3 Love and Athletic Performance

The emotionally charged state of RPL may influence an individual’s functioning in other domains involving the motivation-reward system of the brain. For instance, high-level athletes are intensely focused on the rewarding outcomes of excelling in their sport and winning competitions. When these athletes experience RPL, and their reward-motivation system is activated, their athletic performance may either be enhanced or hampered (Birrer & Morgan, 2010). Although there are positive outcomes associated with RPL, which may spillover to enhance athletic performance, negative outcomes may emerge as well (Reis & Aron, 2008). For example, relationships in this stage may be characterized by jealousy or conflict, causing partners to experience mood swings, anxiety, and depression. These outcomes, whether minor or major, can directly affect an athlete’s performance.

### 1.4 Factors Affecting Athletic Performance

Few researchers have examined the association between RPL and athletic performance, but the impact of non-romantic close relationships has been studied. Donohue and colleagues (2007) examined the influence of coaches, family members, and peers, and concluded that each group has an effect on athletic performance. Coaches foster positive outcomes when they utilize constructive feedback and reinforcement techniques (Bolter, & Weiss, 2013; Langan, Blake, & Lonsdale, 2013). Athletes who perceive their coach’s support and praise as well intentioned have higher self-esteem and experience greater enjoyment in their sport (Smith, 2006; Smith, Smoll, & Cumming, 2006). Correspondingly, athletes perceive themselves as less capable when they are exposed to high levels of criticism and low levels of positive reinforcement from coaches. Family relationships also influence an athlete’s performance. Overly high parental expectations can be a source of stress, anxiety, and hardship for an athlete, which hampers their outlook and athletic success (Appleton, Hall, & Hill, 2010; Sagar & Lavallee, 2010).

Athletes involved in team sports experience a performance boost when team members have positive social interactions, group solidarity, and close friendships (Pugh, Wolff, Gilley, DeFrancesco, Gilley, & Heitman, 2000). Many athletes join and remain involved with sports over the long-term in order to foster and maintain such relationships (Weiss & Ferrer-Caja, 2002). Team competitors additionally experience positive emotions resulting from the release of brain chemicals during their sport, and this positive mood can transfer to teammates (Moll, Jordet, & Pepping, 2010). Team members, who engage in celebratory post-shot behaviors with each other, tend to have better athletic outcomes than teams who do not partake in such rituals. The brain peptide involved in team celebration is oxytocin, which is known as the love hormone because it facilitates bonding.

Romantic partners may influence athletic performance in ways that parallel the impact of coaches, family members, and teammates. For example, partners in new relationships experience elevated oxytocin levels (Moll et al., 2010), which may transfer into the athletic environment and positively impact performance. Taken together, the literature indicates that close relationships benefit an athlete’s performance when they are supportive, and detract from performance when they are critical or demanding. We extend this research by examining the specific influence of romantic relationships on athletic performance, including whether RPL may help or hinder performance.

## 2. Methodology

### 2.1 Participants

Participants were 20 Olympic athletes (19 male, 1 female) from the countries of Belarus, Canada, France, Germany, Norway, the Ukraine, and the United States. The mean age was 28.14 years (Range = 18-33 years.) Nine athletes were competing in ice hockey, 4 in biathlon, 3 in curling, 1 in snowboarding, 1 in aerial skiing, 1 in speed skating, 1 in figure skating, 1 in cross country skiing, and 1 in downhill skiing. Seven participants were married, 4 were cohabiting with a partner, 5 were in a serious relationship, and 4 were not currently in a romantic relationship.

### 2.2 Procedure and Interview Questions

Two researchers attended the Olympic Games and recruited athletes for the study. Participants were approached at entrance points to Olympic Village and public areas surrounding the Village including restaurants and coffee shops. After confirming their status as an Olympic athlete with an ID badge, participants were provided with the study consent form. If they agreed to participate, they were read the following, “Being in-love usually occurs at the beginning of a relationship and involves thinking constantly about the other person, having an intense attraction for them, and wanting to be with them most of the time. Have you ever been in love?” If they responded “yes,” they were read the next question, “Do you think your athletic performance was better or worse while in love? Please explain.” Participants also responded to questions about their age, sport, and relationship status. The researchers took notes during the interviews and wrote additional notes afterwards. The results were grouped by themes and are presented below.

## 3. Results

### 3.1 Are You Currently in Love?

Twenty-two athletes were approached for the study; however, two indicated that they had never been in love. Only those athletes who had been in-love (n=20) were asked to respond to the following question about athletic performance.

### 3.2 Was Your Performance Better or Worse While in Love?

#### 3.2.1 Better

Most participants (n = 15) felt their athletic performance was better while in love. Reasons for those who reported improvements tended to focus on non-RPL explanations. One participant noted that his performance was better because his girlfriend “lives with [him] and helps handle pressure while supporting [him].” Athletes indicated that supportive partners helped manage housework and childcare, which, according to the athletes, freed up extra time for athletic training, and thereby enhanced their performance. As one athlete stated, “My wife helps with chores at home so I have more time for training.” The athletes also commented on the benefits of having a spouse and family (i.e., children) in their lives because these relationships provided added motivation to excel in their sport. As one athlete stated, “It helps to know that my wife and son are in the crowd. I do better because I want my son to be proud of his father;” another indicated, “I have a different point of view since my son was born.”

#### 3.2.2 Unsure

Five participants felt unsure about the effects of RPL on their athletic performance. Despite their uncertainty, these athletes indicated that their sport improved after becoming involved with their partner. They made comments such as, “I am doing better now with my wife in my life, but I think I have become a better athlete during these years so if she was not there, I would be doing the same” and “I am now in a relationship but I have been training a lot more and so I think [my performance] is better because of my training. I’m not sure.” Another participant indicated, “It depends on whether my partner is jealous”; through various relationships (i.e., while in love), he noticed that his athletic performance was enhanced when he was involved with a secure partner, and negatively affected with emotionally taxing or insecure partners. Another participant stated, “Being in love may be better for a professional athlete, but bad for an amateur athlete.” He believed amateur athletes experience unique stressors (e.g., financial strain, lack of formal training) that are less common amongst professional athletes, and that being in love would add to, rather than buffer those stressors.

## 4. Discussion

This study is a first to examine the association between RPL and athletic performance. Our goal was to provide preliminary data that could be used to inform future studies on this topic. Based on Sternberg (1986; 1998) and Fisher’s (2006) theories, and Aron et al.’s (2005) empirical work, we speculated that RPL and athletic performance may involve the reward-motivation system, which could either enhance or detract from experiences in each domain. This research represents a first step in a line of work that is examining the effect of love on athletic performance. Our hope is to ultimately collect biological data. We found that in general, athletes believed their sport was enhanced when in love. However, the reasons provided for improvement were not always related to RPL.

Although athletes had been provided with a definition of RPL at the beginning of the interview, their responses reflected companionate, rather than passionate love. Recall that high levels of intimacy and commitment, rather than passion define companionate love, which makes this love-type more stable and predictable compared to the sexually charged state of RPL (Sternberg, 1998). Responses such as “my wife helps with chores at home so I have more time for training” fit with Western conceptualizations of companionate rather than passionate love. When athletes experience companionate love, the qualities of reinforcement and support may be transferred into the relationship, which could explain why the athletes in our study identified more companionate-related reasons for their athletic success while in love. Previous research reinforces the notion that supportive relationships help bolster performance (Donohue, Miller, Crammer, Cross, & Covassin, 2007). Given that RPL involves intense focus on a partner (Fisher, 2006; Fisher et al., 2005), the relationship may subsume energy that would otherwise be devoted to athletics. This effect may be especially pronounced if the relationship is characterized by jealousy or conflict.

Some participants were unsure whether RPL enhanced their athletic performance. Athletes who are in RPL may feel uncertain because this relationship-state hinders rational thinking. Other side effects of RPL include increased energy, focused attention, and enhanced immune functioning (Fisher, 2006), which could positively impact performance. The athletes may have been uncertain about the source of such effects. One athlete’s comment underscores this point, “I am now in a relationship but I have been training a lot more and so I think [my performance] is better because of my training. I’m not sure.” Another explanation for the athletes’ uncertainty may pertain to the transferability of reward-based satisfaction that results from athletic training. Humans have innate reward-motivation needs that can be satisfied in a variety of ways. Although romantic relationships are a primary source of gratification, people satisfy these needs through other rewarding activities (Xu, Jin, Aron, Wei, Westmaas, & Xuchu, 2012). Athletes may experience similar satisfaction levels from their sport and relationship, but may mistake the source of satisfaction and report uncertainty about the effects of RPL on athletic performance.

These initial findings will lead to additional work on the topic. Researchers might extend this work by examining how individual differences impact the association between love and athletic performance. In terms of personality for example, the association between love and athletic performance may vary depending on the prevalence of certain traits. The trait of neuroticism, which is characterized by anxiety and stress, can be particularly detrimental to intimate relationships, yet individuals high on this trait often excel professionally (Nettle, 2006). High performance athletes who exhibit neurotic tendencies may therefore experience benefits with respect to their sport, but adverse outcomes in their relationships.

Given the pervasive influence of culture, it will be important for researchers to consider participants’ country of origin, and use culturally sensitive measures in subsequent studies on this topic. For example, Wang (1994) found that Italian men report fewer passionate feelings compared to Americans when love is assessed using the North American standardized Passionate Love Scale (PLS). Although RPL exists cross-culturally (e.g., Xu, Aron, Brown, Cao, Feng, & Weng, 2011), the manner in which it is experienced and therefore assessed may vary. Our findings reinforce this point. Participants were provided with a definition of RPL at the start of the study—derived from the Passionate Love Scale—yet their responses reflected companionate love, suggesting differences in their definitions of love. In future work, researchers might define love more broadly (rather focusing exclusively on RPL) to examine the influence of companionate and/or other love types on athletic performance.

Companionate love is a commitment-based bond that typically lacks the high energy and focused attention characteristic of RPL. However, prior empirical work, as well as our own findings suggest that companionate love may enhance athletic performance. Companionate partnerships tend to be more stable than passionate unions and offer the athlete support and encouragement, which helps them focus on training and competition. Donohue et al. (2007) found that compared to peers and coaches, family members had a stronger influence on the performance of high school athletes because these relationships were more intimate. Extrapolating from this research, supportive romantic relationships would likely have a similar positive effect for high performance athletes.

### 4.1 Limitations and Future Research

As with any research, our study contains some noteworthy limitations. First, we decided to use the Olympic Games for participant recruitment because athletes are matched on a variety of characteristics such as performance level, lifestyle (e.g., intensive training), and age. However, some barriers including language and cultural differences hindered our ability to gather detailed interview data. Future work might focus on participants from one culture or country at a time, rather than assessing individuals from multiple locations in a single study. The ethnic diversity of our sample may also be a strength, however, because it highlighted potential variations in cross-cultural definitions of love.

In future studies, researchers could gather quantitative data from a larger sample to allow for an examination of differences based on sport, including team versus individual sports. Research indicates that compared to individual competitors, team athletes experience greater positive emotions and social interactions through their sport (Pugh et al., 2000). RPL may therefore have a greater impact on athletes involved in team sports. Of the 20 participants in our study, 16 were involved in team sports, and of those, 14 reported that their performance was better while in love.

Our study focused on a small group, which prohibited generalization of the findings. The sample also contained a preponderance of men and future work will benefit from the inclusion of more women. Men and women tend to differ in their definitions of love, with women being more likely to describe it as a feeling that unites people (i.e., companionate-focused) and men being more likely to describe it in sexual terms (i.e., passionate-focused) (Borusiak, 2013). Despite these limitations, our study contributes valuable information to the growing body of research on the impact of love across domains. To our knowledge, this study represents a first attempt at understanding the association between RPL and athletic performance. We hope these initial findings will inspire additional work on this topic.
